# The Response to High CO_2_ Levels Requires the Neuropeptide Secretion Component HID-1 to Promote Pumping Inhibition

**DOI:** 10.1371/journal.pgen.1004529

**Published:** 2014-08-07

**Authors:** Kfir Sharabi, Chayki Charar, Nurit Friedman, Inbar Mizrahi, Alon Zaslaver, Jacob I. Sznajder, Yosef Gruenbaum

**Affiliations:** 1 Department of Genetics, Institute of Life Sciences, Hebrew University of Jerusalem, Jerusalem, Israel; 2 Division of Pulmonary and Critical Care Medicine, Feinberg School of Medicine, Northwestern University, Chicago, Illinois, United States of America; University of Washington, United States of America

## Abstract

Carbon dioxide (CO_2_) is a key molecule in many biological processes; however, mechanisms by which organisms sense and respond to high CO_2_ levels remain largely unknown. Here we report that acute CO_2_ exposure leads to a rapid cessation in the contraction of the pharynx muscles in *Caenorhabditis elegans*. To uncover the molecular mechanisms underlying this response, we performed a forward genetic screen and found that *hid-1*, a key component in neuropeptide signaling, regulates this inhibition in muscle contraction. Surprisingly, we found that this *hid-1*-mediated pathway is independent of any previously known pathways controlling CO_2_ avoidance and oxygen sensing. In addition, animals with mutations in *unc-31* and *egl-21* (neuropeptide secretion and maturation components) show impaired inhibition of muscle contraction following acute exposure to high CO_2_ levels, in further support of our findings. Interestingly, the observed response in the pharynx muscle requires the BAG neurons, which also mediate CO_2_ avoidance. This novel *hid-1*-mediated pathway sheds new light on the physiological effects of high CO_2_ levels on animals at the organism-wide level.

## Author Summary

Carbon dioxide (CO_2_) is a key molecule in many biological processes. High levels of CO_2_ in patients with pulmonary diseases are associated with worse outcomes. However, mechanisms by which organisms sense and respond to high CO_2_ levels remain largely unknown. Using *Caenorhabditis elegans* as a model system, we found that exposure to high CO_2_ levels leads to a very rapid cessation in the contraction of the pharynx muscles. Further analysis revealed that the pharynx muscle response is controlled by dense core vesicle secretion from the BAG neurons in a *hid-1*-mediated pathway. This novel *hid-1* pathway sheds new light on the physiological effects of high CO_2_ levels on animals at the organism-wide level.

## Introduction

One of the fundamental features shared by most, if not all, living organisms is the ability to maintain levels of carbon dioxide (CO_2_). Of particular importance is the ability of many animals to sense and respond to high levels of CO_2_ by either attraction or aversion [1]–[5]. In mammals, high levels of CO_2_ (hypercapnia) impair alveolar epithelial function of the lungs by activating the stress sensor AMPK, which leads to Na,K-ATPase endocytosis, impaired cell proliferation, and loss of distal lung epithelial function [6]–[10]. In addition, hypercapnia suppresses specific innate immune responses in *Drosophila* and mice, which increases mortality in a model of pneumonia and leads to changes in gene expression through the NF-kB pathway [11]–[14]. Cyclic AMP (cAMP) signaling also plays a role in the response of mammalian cells to elevated CO_2_ levels [15]–[17]. The molecular pathways mediating the responses to hypercapnia are the focus of intensive research (see [11], [18] and review in [19]).

High levels of CO_2_ quickly elicit an avoidance response in wild-type *Caenorhabditis elegans* animals via a cGMP signaling pathway [2], [4]. The cGMP-regulated avoidance response requires the CO_2_- and oxygen (O_2_)–sensing BAG neurons, in which the guanylyl cyclase receptor, *gcy-9*, controls the response to CO_2_ [20]–[22]. Interestingly, the response to hypercapnia requires the ETS-domain transcription factor, ETS-5, which controls the expression of *gcy-9* in the BAG neurons and plays a role in BAG neuron differentiation [20]–[22]. Recently, the thermosensory AFD neurons and the salt-sensing ASE neurons were also shown to participate in CO_2_ sensing and avoidance [23]. These neurons, however, differ in their response kinetics to high levels of CO_2_; whereas BAG neurons reach maximal activation within 30 s, ASE neurons reach maximal activation only after 2 min, and AFD neurons show intricate dynamics in which Ca^2+^ levels first drop and then increase to maximal levels after 2 min [23]. Interestingly, starved *C. elegans* do not avoid high CO_2_ levels, nor do animals with defects in the *daf-2* signaling pathway, which is an important regulator of the starvation response [2], [4].

In addition to avoidance, *C. elegans* exposed to high CO_2_ levels show specific phenotypes independent of pH [24]. These include a smaller brood size, delayed development, reduced motility coupled with deterioration of striated muscle, and a significant increase in lifespan that is independent of known life-extending pathways [24].

Here we report that exposure of *C. elegans* animals to short (10 s) hypercapnia-inducing levels of CO_2_ (≥5%) leads to a significant reduction in the rate of pharyngeal muscle contraction (pumping). Strikingly, this effect is independent of any currently known molecular pathways that regulate CO_2_ avoidance or O_2_ sensing. Specifically, through a forward genetic screen, we identified a novel participant in the response to CO_2_, *hid-1*, that plays a role in continued pumping in the presence of high CO_2_ levels. Moreover, we show that dense core vesicle secretion pathways in the BAG neurons contribute to the reduced pumping rate in response to high CO_2_ levels.

## Results

### High levels of CO_2_ significantly reduce the pumping rate of the pharynx

**Figure 1:**
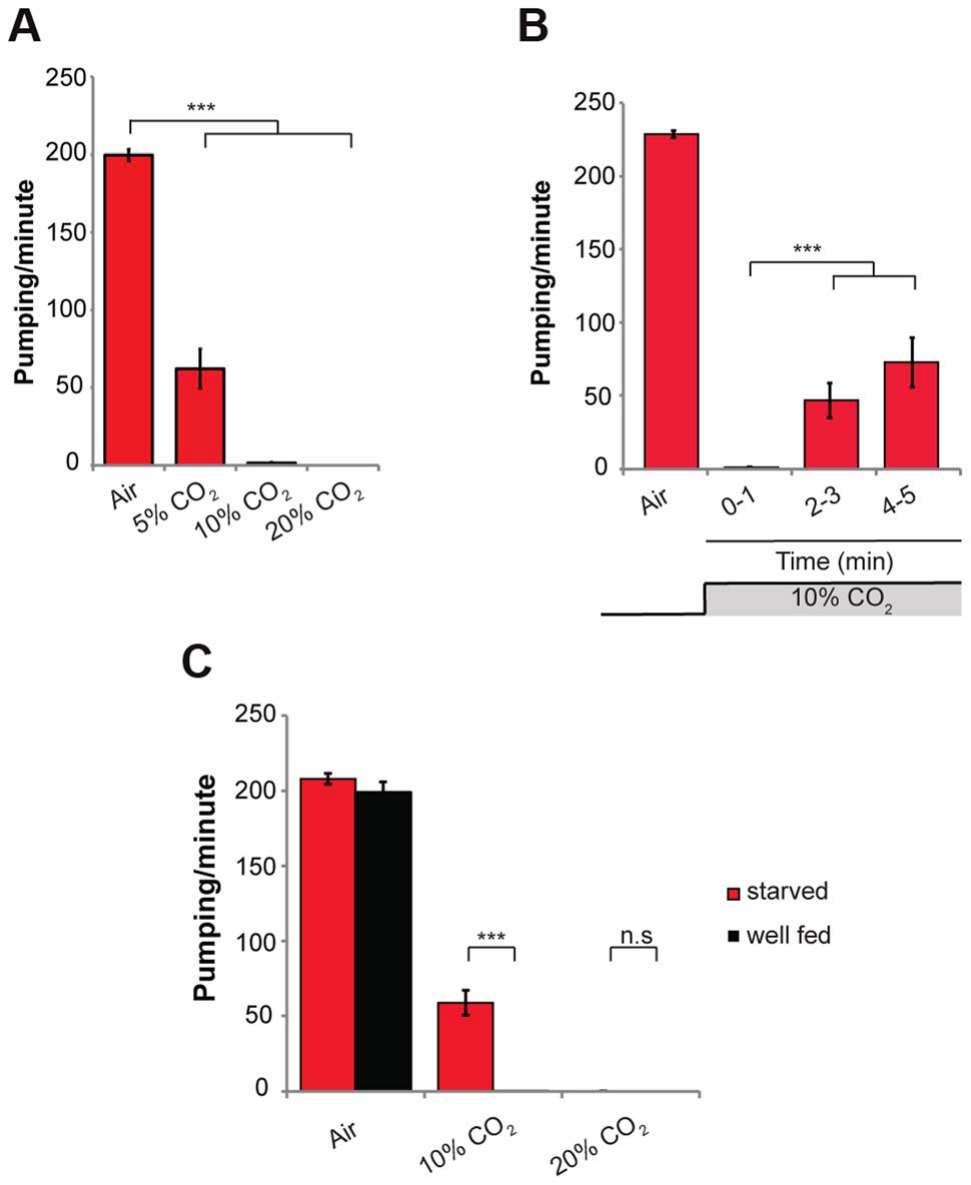
High levels of CO_2_ reduce the pumping rate of the pharynx. (**A**) One-day-old wild-type (N2) adult *C. elegans* were exposed to 5%, 10%, or 20% CO_2_ balanced with 21% O_2_ and N_2_. The pumping rate was measured under a dissecting microscope while the animals were exposed to different gas mixtures. A gas mixture of 21% O_2_ and 79% N_2_ was used as a normal air control. (**B**) One-day-old wild-type (N2) adult *C. elegans* were continuously exposed to 10% CO_2_ for 5 min. The pumping rate was measured during minutes 1, 3, and 5 of exposure to CO_2_. (**C**) One-day-old wild-type (N2) adult *C. elegans* were starved for 4 h and then the pumping rate was measured in 10% or 20% CO_2_. Well-fed worms were used as a control. In all experiments *N*≥30 animals. Different groups were compared by one-way ANOVA followed by *t* test. ***P<.001. Error bars indicate SEM.

To investigate the effects of acute exposure of wild-type *C. elegans* (N2) to high levels of CO_2_, we exposed 1-day-old adult animals grown on standard NGM plates in a small chamber to gas mixtures containing 21% O_2_ and 5%, 10%, or 20% CO_2_ at 22°C. In normal air (0.0391% CO_2_), the rate of muscle contraction of the pharynx was ∼200 pumps/min. Within 10 s of exposure to 5% CO_2_ balanced with 21% O_2_ and 74% N_2_, the pumping rate of the pharynx was reduced from ∼200 to ∼60 pumps/min (Figure 1A). Exposure to 10% and 20% CO_2_ almost completely stopped the pumping of the pharynx (Figure 1A and Movie S1). After 2–3 min of continuous exposure to 10% CO_2_, pumping rate recovered partially to ∼40 pumps/min, and after 5 min of continuous exposure to 10% CO_2_ it recovered to ∼80 pumps/min (Figure 1B), suggesting a separate, existing mechanism that allows for a partial adaptation. Longer exposures of up to 30 min to 10% CO_2_ did not result in full recovery of the pumping rate (Figure S1A). To test whether the effect on the pumping is mediated by a change in the pH of the growth medium due to high CO_2_ levels, we measured the pumping rate of animals using NGM plates buffered to pH of 5.0 and 7.0 in addition to the normally used medium with a pH of 6.0. We did not find any differences between the animals in different growth mediums, both under normal air conditions and after exposure to 10% CO_2_, which suggests that the effect on the pumping is probably not mediated by changes in pH (Figure S1B). This conclusion is supported by a recent finding that activation of CO_2_-responsive neurons can occur independently of changes in extracellular or intracellular acidosis [25]. In addition, mutations in the carbonic anhydrase genes (*cah-2*, *cah-5*, and *cah-6*), which catalyze the conversion of CO_2_ into bicarbonate, had no effect on the pumping rate (Figure S1C). This suggests that the conversion of CO_2_ into bicarbonate is not necessary to induce the response of the pharynx. However, we cannot rule out the possibility of redundancy between the different carbonic anhydrase genes.

The response of the pharynx to high CO_2_ levels was partially dependent on the nutritional state of the animal. Whereas “well fed” animals exposed to 10% CO_2_ stopped pumping, animals starved for 4 h continued pumping at a rate of ∼60 pumps/min (Figure 1C). These starved animals exposed to 20% CO_2_ stopped pumping, similar to wild-type animals (Figure 1C), suggesting a threshold effect of high CO_2_ levels. Together, these data demonstrate that high CO_2_ levels quickly affect muscle contraction of the pharynx, an effect that depends on the nutritional state of the animal.

### The reduction in pumping is independent of CO_2_ avoidance and O_2_ sensing

*C. elegans* animals quickly withdraw when acutely exposed to CO_2_. This response, known as CO_2_ avoidance, is regulated by cGMP signaling [2], [4]. TAX-2 and TAX-4 are two subunits of a cGMP-gated ion channel required for normal chemosensory and thermosensory responses. *C. elegans* null mutants for either TAX-2 *(tax-2[p691])* or TAX-4 *(tax-4[p678])* do not avoid high CO_2_. In addition, the insulin-IGF pathway mediates CO_2_ avoidance, as *daf-2* mutants show reduced CO_2_ avoidance [2], [4]. The avoidance response also requires proper development of ciliated sensory neurons. Animals with mutations in *osm-3* and *che-10* have abnormal cilia as well as defective CO_2_ avoidance. In *C. elegans* strains carrying mutations in *daf-2*, *osm-3*, or *che-10* the CO_2_ avoidance response is either reduced or absent [2], [4].

**Figure 2:**
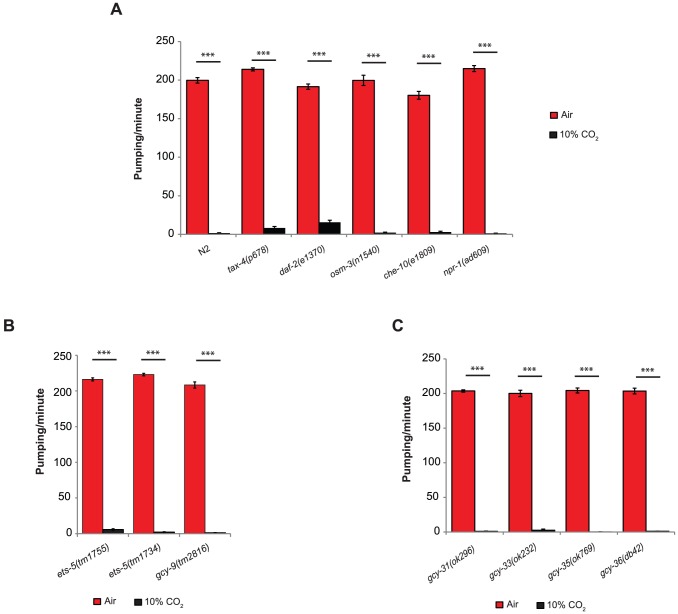
The inhibition of pumping following exposure to high CO_2_ level is independent of molecular pathways that regulate CO_2_ avoidance and oxygen sensing. One-day-old adult *C. elegans* strains containing mutations in genes that regulate CO_2_ avoidance (**A, B**) or O_2_ sensing (**C**) were exposed to 10% CO_2_. The pumping rate was measured under a dissecting microscope during the first minute of exposure to CO_2_. In all experiments *N*≥30 animals. Different groups were compared by one-way ANOVA followed by *t* test. ***P<.001. Error bars indicate SEM.

The effect of high CO_2_ levels on the *C. elegans* pharynx is quick and robust, similar to the avoidance response. However, rather than CO_2_ avoidance, *tax-4(p678)*, *daf-2(e1370)*, *osm-3(n1540)*, and *che-10(e1809)* mutants show a significant reduction in pumping following exposure to 10% CO_2_, similar to the reduction observed in wild-type (N2) animals (Figure 2A). Loss-of-function mutation of the neuropeptide Y receptor, *npr-1*, completely abolishes the CO_2_ avoidance response by inhibiting the activity of the O_2_-sensing URX neurons [3]. In our assay, exposing *npr-1(ad609)* animals to 10% CO_2_ resulted in a response similar to that of wild-type animals (Figure 2A), suggesting that the high activity of the URX neurons in the animals with loss-of-function mutation in *npr-1* does not regulate the pharynx response to CO_2_. The *gcy-9* gene encodes a receptor-type guanylyl cyclase and is a target of the ETS domain ETS-5 transcription factor. Both the *ets-5* and *gcy-9* genes are required for the CO_2_ avoidance response [20]–[22]. When exposed to 10% CO_2_, the *gcy-9(tm2816)*, *ets-5(tm1734)*, and *ets-5(tm1755)* mutants stopped pumping, similar to wild-type animals (Figure 2B), thus further demonstrating that CO_2_ avoidance and acute CO_2_-dependent pumping inhibition are mediated through independent pathways.

To determine whether molecular pathways shared by O_2_ sensing mediate the response of the pharynx to elevated CO_2_ levels, we tested strains with mutations in the *gcy-31*, *gcy-33*, *gcy-35*, or *gcy-36* genes. These genes encode soluble guanylyl cyclases (sGC) that bind O_2_ and sense decreases (*gcy-31* and *gcy-33*) or increases (*gcy-35* and *gcy-36*) in O_2_ levels [26]. Exposing the *C. elegans* strains *gcy-31(ok296)*, *gcy-33(ok232)*, *gcy-35(ok769)*, or *gcy-36(db42)* to 10% CO_2_ resulted in pumping inhibition similar to that observed in wild-type animals (Figure 2C). These results suggest that the effect of high CO_2_ levels on the pumping rate does not involve O_2_ sensing.

### HID-1 is required for the CO_2_-dependent pumping response

**Figure 3:**
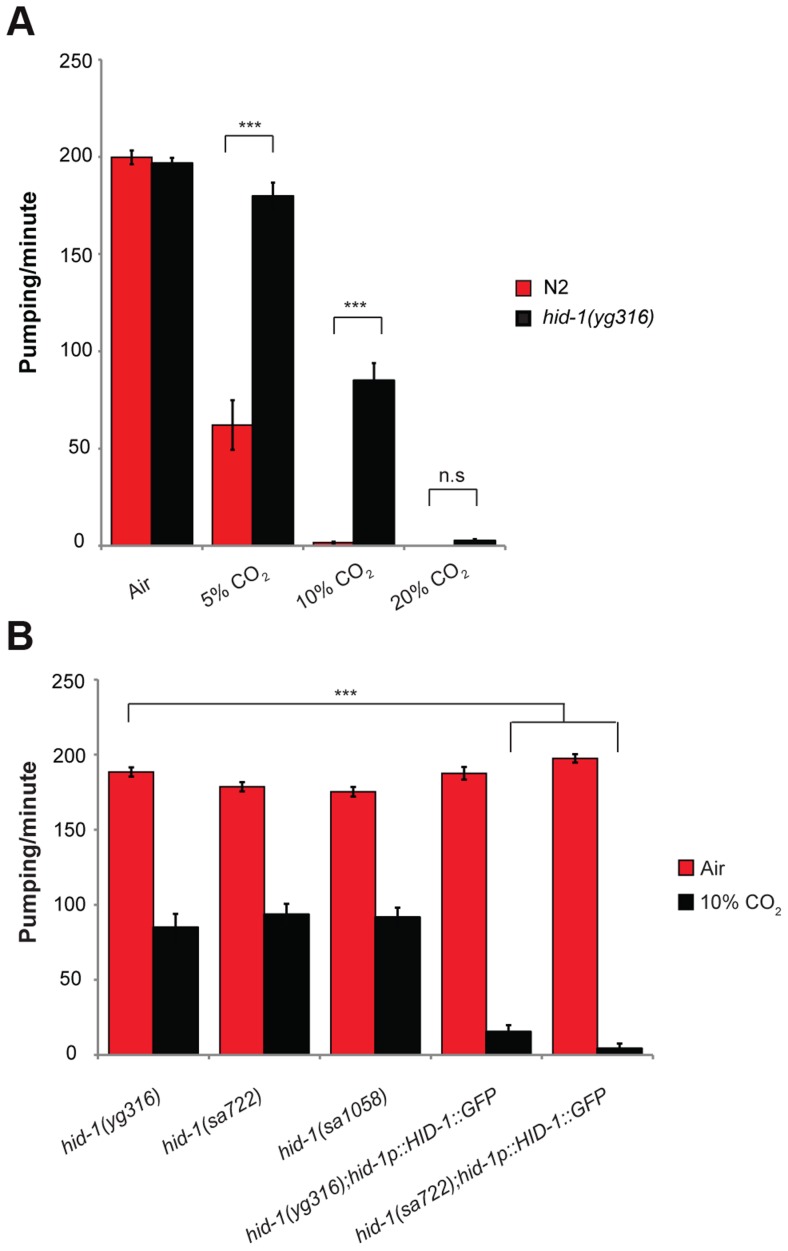
HID-1 is required for sensing CO_2_ level in the pharynx. (**A**) One-day-old adult *hid-1(yg316)* and N2 worms were exposed to 5%, 10%, or 20% CO_2_ balanced with 21% O_2_ and N_2_. The pumping rate was measured under a dissecting microscope while the animals were exposed to the different gas mixtures. A gas mixture of 21% O_2_ and 79% N_2_ was used as a normal air control. (**B**) The inhibition of the pumping rate of the pharynx after exposure to high CO_2_ level in *hid-1(yg316)* allele mutants is significantly reduced. Similarly, the inhibition of the pumping rate of the pharynx after exposure to high CO_2_ level is reduced in other *hid-1* allele mutants (*sa772* and *sa1058*). Transgenic expression of HID-1 fused to eGFP in the *sa722* or *yg316* background (*hid-1(sa722*);HID-1::GFP or *hid-1(yg316)*;HID-1::GFP) is sufficient to restore the effect of high CO_2_ level on the pumping rate back to the wild-type phenotype. In all experiments *N*≥30 animals. Different groups were compared by one-way ANOVA followed by *t* test. ***P<.001. Error bars indicate SEM.

To identify genes that are involved in regulating the pharynx response to 10% CO_2_, we performed a forward genetic screen after ethyl methanesulfonate (EMS) mutagenesis. Specifically, we screened for mutant animals that do not stop pumping in response to 10% CO_2_ (Movie S2). We screened the progeny of ∼1200 F1 animals and found three strains that continued pumping when exposed to 10% CO_2_. One of these strains was further crossed to the Hawaiian strain, and deep sequencing was performed on DNA from recombinant F2 progeny. The region containing the mutant gene that enabled continuous pumping in 10% CO_2_ was identified by searching for a low number of Hawaiian single-nucleotide polymorphisms (SNPs), as described elsewhere [27]. This mutant strain has a premature stop codon in a previously characterized highly conserved gene, *hid-1*. In 5% CO_2_, unlike in wild-type animals, the pumping rate of animals with the isolated *hid-1*(*yg316*) allele was similar to the pumping rate in normal air conditions, and significant pumping continued after exposure to 10% CO_2_, whereas in 20% CO_2_ pumping was abolished (Figure 3A).

The effect of HID-1 on the response to high levels of CO_2_ was specific to the pharynx, since *hid-1* mutant animals still showed reduced egg laying (Figure S2) and a slower rate of development (data not shown), similar to wild-type animals exposed to high CO_2_ levels [24]. Two other alleles of *hid-1*, *sa722* and *sa1058* [28], also showed continuous pumping when exposed to 10% CO_2_ (Figure 3B). The change in pumping rate in response to CO_2_ is specific to HID-1, since transgenic expression of HID-1::GFP under its own promoter, in either *hid-1(sa722)* or *hid-1(yg316)* strains (Figure S3), was sufficient to restore the normal reduced pumping rate in 10% CO_2_ (Figure 3B). Together, these data suggest that *hid-1* is required for the response of the pharynx to high levels of CO_2_.

### Other dense core vesicle secretion and maturation mutants are also involved in the inhibition of pumping by CO_2_

**Figure 4:**
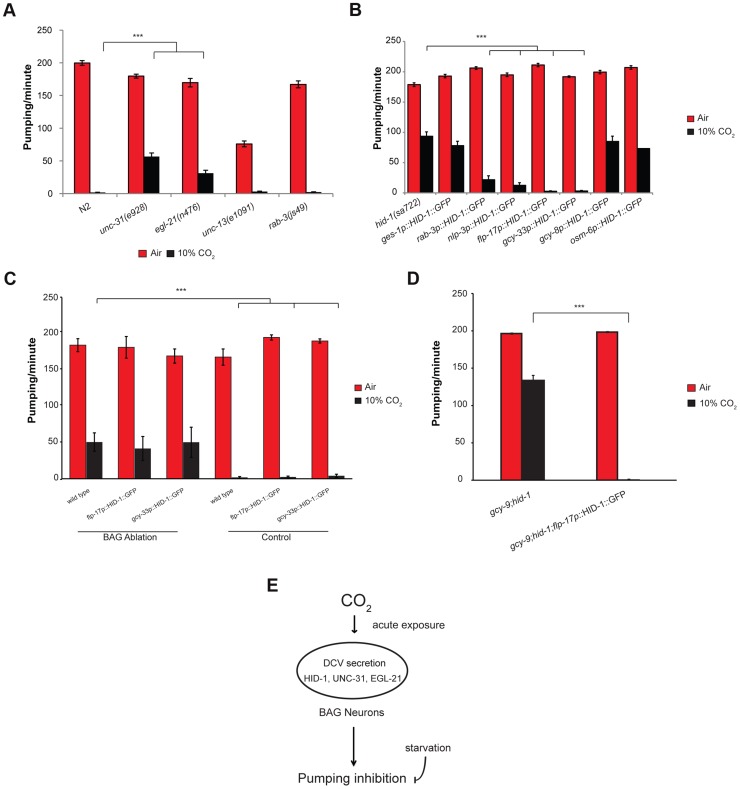
The effect of high CO_2_ level on the pharynx requires HID-1 activity in the BAG neurons. (**A**) One-day-old adult *C. elegans* strains containing mutations in *unc-31* or *egl-21* genes, which are important for proper neuropeptide secretion and maturation, show a significant rescue of the pumping rate after exposure to 10% CO_2_. In contrast, strains with mutations in *unc-13* or *rab-3*, which promote synaptic vesicle secretion, do not show a changed pharynx response to 10% CO_2_. (**B**) Transgenic expression of HID-1 in the gut using the gut-specific *ges-1* promoter (*ges-1p*-HID-1::GFP) was not sufficient to restore pumping phenotype to wild type after exposure to 10% CO_2_. In contrast, transgenic expression of HID-1 in neurons using the *rab-3* promoter (*rab-3p*-HID-1::GFP) was sufficient to restore pumping rate after exposure to 10% CO_2_ almost back to wild-type levels. Cell-specific expression of HID-1 in the AFD neurons (*gcy-8p*-HID-1::GFP) or in the amphid and tail ciliated neurons, including ASE neurons (*osm-6p*-HID-1::GFP), did not restore the CO_2_ effect on the pumping back to wild-type levels, whereas cell-specific expression of HID-1 in sensory and pharyngeal neurons (*nlp-3p*-HID-1::GFP) or in BAG neurons (*flp-17p*-HID-1::GFP and *gcy-33p*-HID-1::GFP) was sufficient to restore the effect of high CO_2_ level back to the wild-type phenotype. (**C**) The BAG neurons of wild-type *C. elegans* expressing *gcy-33*::GFP were laser ablated and the pharyngeal pumping rate was measured in normal air and 10% CO_2_. Similarly, the BAG neurons of *flp-17*p::HID-1::GFP and *gcy-33*p::HID-1::GFP strains were laser ablated and the pharyngeal pumping rate subsequently measured. Controls include measurement of the pumping rate in the same *C. elegans* strains without ablation. (**D**) Transgenic expression of HID-1 in the BAG neurons of *hid-1(sa722);gcy-9(tm2816)* animals restores the suppression of pumping in the presence of high CO_2_ level. (**E**) Schematic model of CO_2_ response of pharyngeal muscle contraction. The inhibition of muscle contraction in the pharynx is mediated by neuropeptide secretion via dense core vesicles (DCVs) in BAG neurons. The CO_2_ response is decreased after starvation. In all experiments *N*≥30 animals, except in panel C in *flp-17p*::HID-1::GFP (*N* = 5) and *gcy-33p*::HID-1::GFP (*N* = 10). Different groups were compared by one-way ANOVA followed by *t* test. ***P<.001. Error bars indicate SEM.

Dense core vesicles (DCVs) secrete neuropeptides in peptidergic neurons [29]. HID-1 is associated with Golgi membranes by way of N-terminal myristoylation and is required for the sorting of DCVs, where it prevents sorting of peptide cargoes to lysosomes for degradation [30]–[32]. We hypothesized that HID-1 plays a role in the response of the pharynx to high CO_2_ by regulating neuropeptide secretion. We tested this hypothesis by scoring pumping response to 10% CO_2_ in mutants defective in other genes involved in neuropeptide secretion. The gene *unc-31* encodes the *C. elegans* ortholog of CAPS (calcium-dependent activator protein for secretion), an important component of DCV exocytosis [33]. The gene *egl-21* encodes the *C. elegans* ortholog of carboxypeptidase E, an important component in neuropeptide maturation [34]. Following exposure of *unc-31(e928)* or *egl-21(n476)* deletion strains to 10% CO_2_, the pumping rate of the pharynx was significantly higher compared with that in wild-type animals exposed to the same concentration of CO_2_ (Figure 4A). We also tested the role of synaptic vesicle secretion on the pumping response to 10% CO_2_. The *unc-13* gene is involved in synaptic vesicle secretion of neurotransmitters [35], [36]. The *rab-3* gene is a Rab GTPase that affects the distribution of synaptic vesicle populations [37]. Exposure of *unc-13(e1091)* or *rab-3(js49)* mutant strains to 10% CO_2_ showed pumping behavior similar to that of wild-type strains (Figure 4A). These data suggest that DCVs play an important role in mediating the response of the pharynx to high CO_2_ levels and that compromising DCV secretion probably impairs the pumping response to high CO_2_ levels.

### Expression of HID-1 in the BAG neurons is sufficient to restore wild-type CO_2_ response in *hid-1* mutant strains

HID-1 is expressed in all neuron and gut cells of *C. elegans* [30]. To test whether inhibition of pharynx pumping in response to 10% CO_2_ requires expression of *hid-1* in the gut, neurons, or both, we used transgenic lines that express HID-1 fused to GFP driven by either the pan-neuronal promoter *rab-3* or the gut-specific promoter *ges-1*. Expression of HID-1 under the *rab-3* promoter in neurons of *hid-1*(*sa722*) background was sufficient to restore inhibition of the pharynx pumping almost to the levels shown by wild-type animals (Figure 4B). In contrast, expression of HID-1 under the *ges-1* promoter in the gut of *hid-1*(*sa722*) had no significant effect on the response of the pharynx to 10% CO_2_.

We next asked which subset of neurons is required for mediating the effect of high CO_2_ levels on the pharynx. The *nlp-3* gene is expressed in sensory neurons (ADF, ASE, ASH, AWB, ASJ, and BAG) as well as in pharyngeal neurons (I1, I2, I3, I4, M1, M3, and NSMR) (Figure S3) [38]. Transgenic expression of HID-1::GFP under the *nlp-3* promoter in *hid-1*(*sa722*) background was sufficient to restore pharynx pumping inhibition after exposure to 10% CO_2_ (Figure 4B). High levels of CO_2_ activate the AFD neurons [23]. Surprisingly, transgenic expression of HID-1::GFP driven by a *gcy-8* promoter in the thermosensory AFD of *hid-1*(*sa722*) background did not restore the CO_2_-mediated pumping inhibition (Figure 4B), which suggests that the activation of the AFD neurons by high CO_2_ levels is not sufficient to induce the peptidergic signaling that mediates the effect of high CO_2_ levels on the pharynx. Among the sensory neurons expressing *nlp-3* are the BAG and the ASE neurons, which are also known to respond to high CO_2_ levels [23]. Transgenic expression of HID-1::GFP driven by the *osm-6* promoter, which was expressed in ASE neurons (undetected in BAG neurons), did not restore the CO_2_-mediated pumping inhibition (Figure 4B). We next tested the role of BAG neurons in the CO_2_-dependent pumping inhibition of the pharynx. Transgenic lines expressing HID-1::GFP under the promoter of *flp-17* showed expression in the BAG neurons (Figure S3). This expression was sufficient to fully restore the CO_2_-dependent pumping inhibition (Figure 4B). Similarly, the expression of HID-1::GFP under the *gcy-33* promoter in *hid-1*(*sa722*) background was specific to the BAG neurons (Figure S3) [21]. This expression was sufficient to fully restore the CO_2_-dependent pumping inhibition (Figure 4B). Next, we ablated the BAG neurons in transgenic worms expressing HID-1::GFP under *flp-17* and *gcy-33* promoters in *hid-1*(*sa722*) background. We found that following the removal of the HID-1::GFP-expressing BAG neurons, the pumping in 10% CO_2_ was similar to that of *hid-1*(*sa722*) animals (Figure 4C). These results suggest that the specific expression of HID-1 in the BAG neurons is sufficient to induce the CO_2_-dependent pumping inhibition. We also ablated the BAG neurons in wild-type background using GFP driven by *gcy-33* promoter as a marker. We found that following ablation of the BAG neurons, the pumping in response to 10% CO_2_ was similar to that in HID-1-null animals (Figure 4C). These results suggest that the BAG neurons are required for the pumping inhibition. In addition, to test possible cross talk between the neuropeptide secretion pathway and the guanylyl cyclase receptor pathway, which is required for CO_2_ avoidance, we measured the pharyngeal pumping rate of animals carrying both *hid-1*(*sa722*) and *gcy-9(tm2816)* mutations. The pumping rate was similar to that of *hid-1*(*sa722*) animals (Figure 4D). Moreover, in the same genetic background, transgenic expression of HID-1 in the BAG neurons, using the *flp-17* promoter, restored the suppression of pumping in the presence of high CO_2_ level (Figure 4D), further demonstrating that the response to CO_2_ mediated by *hid-1* is independent of the response to CO_2_ mediated by *gcy-9*. We conclude that proper *hid-1* activity in the BAG neurons is important to mediate the pumping inhibition by CO_2_.

## Discussion

In humans, high CO_2_ levels have diverse effects on the lung epithelium, immunity, and muscle function. However, the effects of acute exposure of muscle cells to high CO_2_ levels were unknown. In addition, recent studies suggest that mammals, like *C. elegans*, are able to sense elevated CO_2_ levels, which is of broad physiologic significance.

### CO_2_ avoidance and CO_2_-dependent reduced pharyngeal pumping are probably regulated via different pathways

Acute exposure of well-fed adult *C. elegans* animals to high CO_2_ levels quickly reduces the pumping rate of the pharynx. This effect depends in part on the nutritional status of the animal, since starved animals exposed to 10% CO_2_ in air continue to pump, albeit at a significantly slower rate. Our genetic data suggest that the effect of acute exposure to high CO_2_ levels on the pumping rate is independent of the avoidance responses of *C. elegans* to high CO_2_ levels. First, cGMP signaling is required for mediating the avoidance response, as mutations in the cGMP gated ion channel encoded by *tax-2* and *tax-4* completely disrupt the avoidance response [2], [4]. In contrast, the same mutation in *tax-4* does not completely rescue the immediate response of the pumping rate to high CO_2_ levels. Second, mutation in the insulin-like receptor encoded by *daf-2* also disrupts the avoidance response [2], [4]. The pumping rate of *daf-2* mutants under exposure to 10% CO_2_ is dramatically reduced, like in the wild-type animals. The limited recovery of the pumping rate in *daf-2* mutants at 10% CO_2_ could be due to the effect of *daf-2* on starvation regulating pathways [39], [40]. Third, interference with proper function of ciliated sensory neurons by mutations in *osm-3* and *che-10* also significantly changes the avoidance response. Again, in the pumping assay, similar mutations in these genes did not change the response of *C. elegans* to high CO_2_ levels. In addition, mutations in *ets-5* and *gcy-9*, which were previously shown to be required for the calcium response of the BAG neurons to CO_2_, did not change the response of the pharynx to high CO_2_ levels. Finally, the rescue of pumping by *hid-1* in the BAG neurons was not affected by *gcy-9* mutations.

*C. elegans* animals presumably interpret high CO_2_ levels as a harmful cue that leads to avoidance and pumping inhibition. The ability of the animal to stop eating for several minutes probably allows it to avoid undesirable food. Surprisingly, although both the avoidance and the pumping responses to the same stressful cue are immediate, our genetic data suggest that different molecular pathways mediate the two responses to high CO_2_.

### The potential role of neuropeptides in the response of the pharynx to high levels of CO_2_

Our genetic screen identified *hid-1* as a regulator of the pumping response to high CO_2_ levels, as mutations in the *hid-1* gene blunted the response of the pharynx to high CO_2_ levels. HID-1 is required for the neuropeptide secretion pathway [30], [32]. Indeed, mutations in other known genes in peptidergic signaling, *unc-31* and *egl-21*, could also partially suppress the pharyngeal pumping suppression upon exposure to 10% CO_2_. Neuropeptides are important signaling molecules in many physiological responses both in *C. elegans* and in other organisms. In *C. elegans* there are more than 250 neuropeptides that play a role in feeding and metabolism, and most neurons in *C. elegans* secrete neuropeptides [41]. Neuropeptides are also secreted from the intestine [38], and *hid-1*, an important peptidergic signaling gene, is expressed both in the nervous system and in the intestine [30], [32]. Neuropeptide signaling was previously shown to regulate pumping inhibition in the absence of food [42]. Specifically, *unc-31* mutants demonstrate continuous pumping in the absence of food, unlike wild-type animals [42]. Since *hid-1* also partially suppresses the inhibition of pumping in the absence of food (data not shown), we cannot completely rule out the possibility that *hid-1* generally inhibits pumping and acts in parallel with CO_2_.

The pharynx response to 10% CO_2_ is probably mediated by several different neuropeptides, since pumping inhibition could not be inhibited by deletion of individual neuropeptide genes known to be overexpressed in the BAG neurons, including *flp-10*, *flp-16*, *flp-27*, *nlp-1*, *flp-17*, and *nlp-14* (Figure S4). Neuropeptide secretion can only partially explain the response of the pharynx to high levels of CO_2_, since none of the peptidergic signaling mutants we examined at 10% CO_2_ (*hid-1*, *unc-31*, and *egl-21*) exhibited the pumping rate seen at normal air levels (Figure 4). Thus we cannot completely rule out the possibility that the effects of *unc-31* and *egl-21* are due to the other pathway(s) that must be acting in parallel with *hid-1*. This implies the existence of other, HID-1-independent mechanisms that must regulate the response of the pharynx to CO_2_ levels. For example, it is possible that high CO_2_ levels trigger other presynaptic inputs that mediate the effect on the pharynx in parallel with the peptidergic signaling, or that CO_2_ has also a direct postsynaptic effect on the pharyngeal muscles that inhibits their normal function. Interestingly, such parallel pathways depend on the CO_2_ levels, since *hid-1* completely rescues the pumping inhibition at 5% CO_2_ and fails to rescue the pumping at 20% CO_2_ (Figure 3A).

### The presence of HID-1 is specifically required in the BAG neurons

Using transgenic lines that express HID-1 either in the gut or in the nervous system we have determined that the *hid-1* activity is specifically required in neurons to mediate the effect of high CO_2_ levels on the pharynx. We used an AFD-specific promoter to show that *hid-1* activity in the AFD neurons, which are activated by high CO_2_ levels [23], is not sufficient to mediate the effect of high CO_2_ levels on the pharynx. In contrast, transgenic expression of HID-1::GFP under the *nlp-3* promoter is sufficient to restore CO_2_-mediated pharynx pumping inhibition. Using the BAG-specific promoters *flp-17* and *gcy-33* and performing ablation experiments on the BAG neurons, we have further narrowed the CO_2_ effect on the pharynx to the BAG neurons. Paradoxically, our genetic data (Figures 2 and 4) suggest the existence of different molecular pathways for the avoidance and the pharynx responses. However, the BAG neurons in the pharynx response are the same neurons that control the avoidance response. Interestingly, mutant animals that block CO_2_-mediated calcium response in the BAG neurons still show normal pumping inhibition. The existence of such a pathway is especially surprising given that DCV secretion is expected to depend on an increase in calcium levels.

The physiological and molecular effects of high CO_2_ levels, in both vertebrates and invertebrates, have been the focus of several recent studies [2], [4], [6], [7], [12], [14], [22], [24], [43]. However, the sensing mechanism of cells to high CO_2_ levels is yet largely unknown. Soluble adenylyl cyclases are bicarbonate sensors in several organisms including mammals [15], [44], [45]. In *C. elegans*, which do not have soluble adenylyl cyclases, the soluble guanylyl cyclases GCY-31 and GCY-33 are important for eliciting CO_2_ avoidance in the BAG neurons [23]. However, it is yet unknown whether the *gcy* genes are directly activated by either CO_2_ or HCO_3_^−^_._ Our results show that neither GCY-31 nor GCY-33 are required for mediating the effect of high CO_2_ levels on the pharynx (Figure 2C).

Our study sheds new light on the response of *C. elegans* to high CO_2_ levels. It also shows that the CO_2_-induced response is differentially regulated across different tissues. Furthermore, different levels of CO_2_ lead to various outcomes in the same tissue. Deciphering the mechanisms underlying these fundamental pathways will hopefully help us to better understand the CO_2_-induced responses that are activated in human diseases.

## Materials and Methods

### Strains

Worms were handled as described elsewhere [46]. The following strains were used in this study: N2 (wild type); CF1041, *daf-2(e1370)*; CB3329, *che-10(e1809*); CX2948, *tax-4(p678)*; PR802, *osm-3(n1540)*; DA609, *npr-1(ad609)*; CZ3714, *gcy-31(ok296)*; CZ3715, *gcy-33(ok232)*; CX6448, *gcy-35(ok769)*; AX1296, *gcy-36(db42)*; JT722, *hid-1(sa722)*; JT1058, *hid-1(sa1058)*; YG316, *hid-1(yg316)*; YG2310, *hid-1(yg316)*; jsEx896 [*hid-1p*::HID-1::GFP]; NM3017, *hid-1(sa722)* and *lin-15(n765)*; jsEx896[*hid-1p*::HID-1::GFP]; NM3053, *hid-1(sa722)* and *lin-15(n765)*; jsEx897[*rab-3p*::HID-1::GFP]; NM3139, *hid-1(sa722)* and *lin-15(n765)*; jsEx909[*ges-1p*::HID-1::GFP]; YG2313, *hid-1(sa722)*; ygEx317 [*gcy-8p*::HID-1::GFP]; YG2318, *hid-1(sa722)*; ygEx318 [*nlp-3p*::HID-1::GFP]; YG2319, *hid-1(sa722)*; ygEx319 [*flp-17-p*::HID-1::GFP]; YG2340, *hid-1(sa722)*; ygEx320 [*gcy-33-p*::HID-1::GFP]; DA509, *unc-31(e928)*; KP2018, *egl-21(n476)*; YG2302, *unc-13*(*e1091*); YG2320, *ets-5(tm1734)*; YG2321, *ets-5 (tm1755)*; YG2322, *gcy-9(tm2816)*; YG2323, *gcy-9(tm2816)* and *hid-1(sa722)*; YG2324, *gcy-9(tm2816)* and *hid-1(sa722)*; ygEx321 [*flp-17-p*::HID-1::GFP]; RB1340, *nlp-1(ok1469)*; RB2575, *flp-19(ok3587)*; VC2012, *flp-27(gk3331)*; VC1108, *nlp-14(ok1517)*/szT1 X; RB1989, *flp-10(ok2624)*; RB2275, *flp-16(ok3085)*. All strains were obtained from the *C. elegans* Genome Center (CGC) or the National BioResourse Project (NBRP), except for CX2948, which was kindly provided by the De-Bono laboratory, and NM3017, NM3053, and NM3139, which were kindly provided by the Nonet laboratory [2], [30].

### Measurement of pumping rate

A standard NGM plate covered with a lid-shaped chamber with inlet and outlet holes to allow gas flow was used to measure the pumping rate under different concentrations of CO_2_ in air. The chamber was connected to a mechanical valve that controlled the humidified gas mixture entering the chamber. For all pumping assays, NGM plates were seeded with 20 µL of OP50 5 h before the experiment to allow normal feeding and to keep worms in a restricted area. A single 1-day-old adult worm was seeded on a plate just before the start of the experiment. Initially, normal air mixture (21% O_2_, 79% N_2_) flowed into the chamber and worms were allowed to adjust for 1 min. The number of pharynx muscle contractions was subsequently measured for 1 min under normal air conditions. Then the airflow was switched to a high-CO_2_ gas mixture, and after 10 s the pharynx muscle contraction rate was measured again. To measure pumping rate after starvation, well-fed wild-type 1-day-old adult worms were collected using M9 buffer and washed four or five times in M9 buffer. Worms were then seeded on either NGM plates with no bacteria or NGM plates seeded with OP50, for 4 h prior to measurements. All pumping assays were performed at 22°C.

### EMS screen and SNP mapping

The EMS mutagenesis was performed essentially as described elsewhere [46]. Briefly, wild-type (N2) worms in the L4 stage were exposed to 50 mM EMS in M9 buffer for 4 h and then transferred to fresh plates for 2–3 h (P_0_) for recovery. After recovery, five P_0_ animals were transferred again to an NGM plate and allowed to lay F1 progeny. Adult F1 animals were cloned onto individual NGM plates and their L4-adult F2 progeny where exposed to 10% CO_2_. F2 worms that continued the pumping of the pharynx even after exposure to 10% CO_2_ were isolated. In total, we scored the progeny of ∼1200 F1 animals. The isolated strains were outcrossed three times. The mutation was mapped as described elsewhere [27]. Mutant worms were crossed with the Hawaiian strain and F1 progeny were isolated. Then 44 F2 recombinants that continued the pumping after exposure to 10% CO_2_ were isolated, and the DNA of their F3 and F4 offspring was extracted using a Gentra Puregene kit (Qiagen, cat. no. 158667). Whole genome sequencing was performed using the Applied Biosystems SOLiD 3 deep sequencing apparatus. The positions of the Hawaiian SNPs were mapped on the DNA of the *yg316* strain. A 1.2-MB region in chromosome X that did not contain any Hawaiian SNP was found. Within this region a premature stop codon (W625X) in the coding sequence of *hid-1* was found to cause the phenotype as described in the text.

### Plasmid constructs, transgenes, and laser ablation

The NM1699 construct, which contains the *hid-1* promoter driving the genomic *hid-1* coding region fused to eGFP, was a kind gift from the Nonet laboratory [30]. The NM1699 construct was digested with KpnI and AatII to replace the native *hid-1* promoter with various neuron-specific promoters. To drive AFD-specific expression an 800-bp fragment upstream of the *gcy-8* start codon was amplified and subsequently digested with KpnI-AatII to generate pKS10. Similarly, to drive sensory and pharyngeal specific expression, a 700-bp fragment upstream of the *nlp-3* start codon was amplified and digested with KpnI-AatII to generate pKS20. To drive BAG-specific expression a 3.4-kb fragment upstream of the *flp-17* start codon was amplified and fused by PCR to HID-1::GFP from NM1699. In addition, to drive BAG-specific expression a 980-bp fragment upstream of the *gcy-33* start codon was amplified and subsequently digested with KpnI-AatII to generate pKS30. All plasmids were verified by sequencing and microinjected to either JT722 or YG316 with an *elt-2*::GFP marker as described elsewhere [47].

Laser ablation was performed using an Andor Revolution XD confocal spinning disk system with a Nikon TiE inverted microscope equipped with a nitrogen pulsed laser and a 365-nm Micropoint dye cell. The microscope and laser were controlled by means of IQ software and the Micropoint Mosaic I System 85-75, respectively. The region of interest was set according to the size of the neuron cell body as revealed by the GFP marker. We used a frequency of 15 Hz, an energy range of 80%–90%, and 3–5 repeats in order to completely ablate the GFP marker in the neuron cell body.

## Supporting Information

Figure S1 Pumping inhibition is not rescued by either 30 min of exposure to 10% CO_2_, pH of 5.0 or 7.0, or mutations in the carbonic anhydrase genes. (**A**) One-day-old wild-type (N2) adult *C. elegans* were continuously exposed to 10% CO_2_ for 30 min and pumping rate was measured at different time points. (**B**) One-day-old wild-type (N2) adult *C. elegans* were transferred to NGM plates buffered at pH of 5.0, 6.0, or 7.0 followed by exposure to 10% CO_2_ and measurements of the pharyngeal pumping. (**C**) One-day-old adult worms with mutations in *cah-2*, *cah-5*, or *cah-6* genes exposed to 10% CO_2_ showed pharyngeal pumping rate similar to that of wild-type animals.
(PDF)

Figure S2 The egg-laying rate of *hid-1(yg316)* animals exposed to 10% CO_2_ is similar to that of wild-type animals. Gravid animals were exposed to either normal air conditions or air containing 19% or 10% CO_2_ for 6 h. The number of embryos laid during this period was measured.
(PDF)

Figure S3 Transgenic expression of HID-1::GFP. HID-1 fused to eGFP was expressed under its own promoter in the background of *yg316* or under *gcy-8*, *nlp-3*, *osm-6*, *flp-17*, or *gcy-33* promoters in the background of *sa722*. Arrows indicate the AFD neurons (*gcy-8p*) and BAG neurons (*flp-17p* and *gcy-33p*).The expression of *hid-1p*, *nlp-3p* and *osm-6p* was detected in several neurons. Scale bar, 10 µm.
(PDF)

Figure S4 Animals with deletions in neuropeptide genes expressed in the BAG neurons still show strong CO_2_-mediated pumping inhibition. One-day-old animals with mutations in neuropeptide genes, which are known to be overexpressed in the BAG neurons, were exposed to 10% CO_2_ and pumping rate was measured. The pumping rate of *nlp-1*, *nlp-14*, and *flp-16* mutants in 10% CO_2_ (but not in normal air conditions) was significantly different from that of the wild-type (N2) animals and showed small but significant rescue. *P<.01. Error bars indicate SEM.
(PDF)

Movie S1 Pumping of wild-type *C. elegans* exposed to 10% CO_2_. Pumping rate of wild-type animal under dissecting microscope is presented. Worms are first exposed to normal air and then exposed to 10% CO_2_.
(AVI)

Movie S2 Pumping of *hid-1(yg316)* mutant exposed to 10% CO_2_. Pumping rate of *hid-1(yg316)* mutant animal under dissecting microscope is presented. Worms are first exposed to normal air and then exposed to 10% CO_2_.
(AVI)
