# Supplementary figures and images for: The Response to High CO2 Levels Requires the Neuropeptide Secretion Component HID-1 to Promote Pumping Inhibition
Source: PLoS Genet. 2014 Aug 7;10(8):e1004529. doi: 10.1371/journal.pgen.1004529 (PMC4125093; doi:10.1371/journal.pgen.1004529)

A

Figure S1

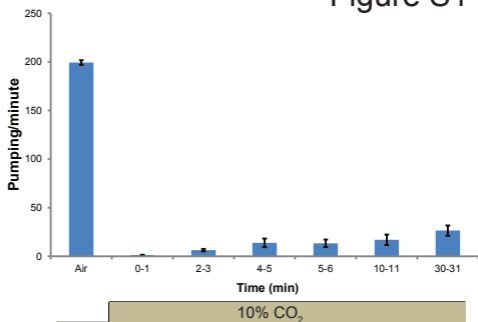

B

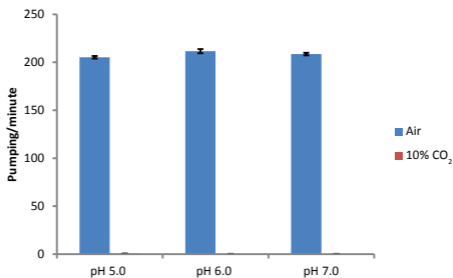

C

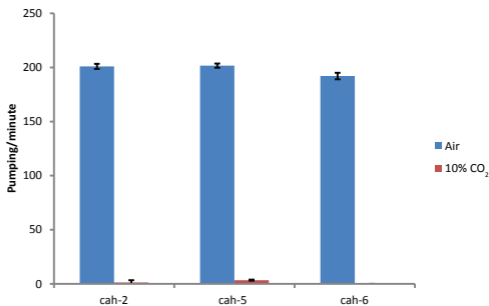

Supplement: Figure S1 — Pumping inhibition is not rescued by either 30 min of exposure to 10% CO2, pH of 5.0 or 7.0, or mutations in the carbonic anhydrase genes. (A) One-day-old wild-type (N2) adult C. elegans were continuously exposed to 10% CO2 for 30 min and pumping rate was measured at different time points. (B) One-day-old wild-type (N2) adult C. elegans were transferred to NGM plates buffered at pH of 5.0, 6.0, or 7.0 followed by exposure to 10% CO2 and measurements of the pharyngeal pumping. (C) One-day-old adult worms with mutations in cah-2, cah-5, or cah-6 genes exposed to 10% CO2 showed pharyngeal pumping rate similar to that of wild-type animals. (PDF) [file pgen.1004529.s001.pdf]

Figure S2

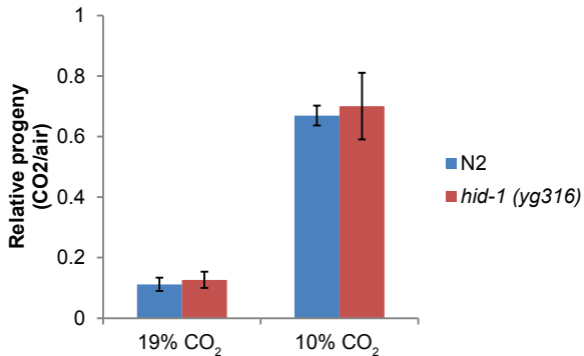

Supplement: Figure S2 — The egg-laying rate of hid-1(yg316) animals exposed to 10% CO2 is similar to that of wild-type animals. Gravid animals were exposed to either normal air conditions or air containing 19% or 10% CO2 for 6 h. The number of embryos laid during this period was measured. (PDF) [file pgen.1004529.s002.pdf]

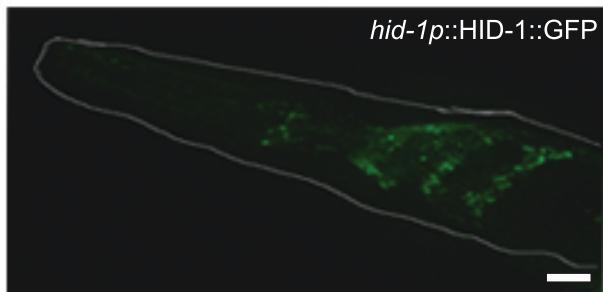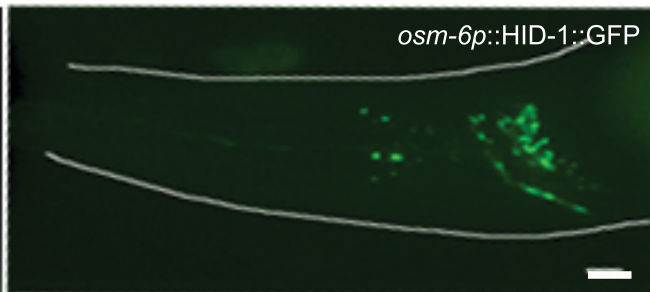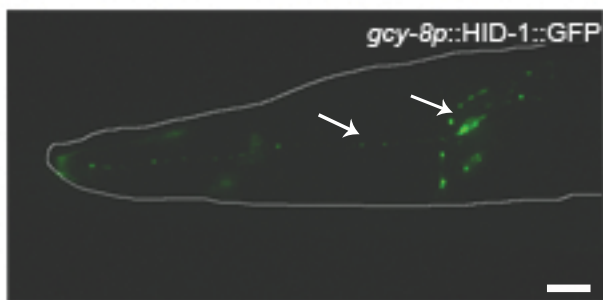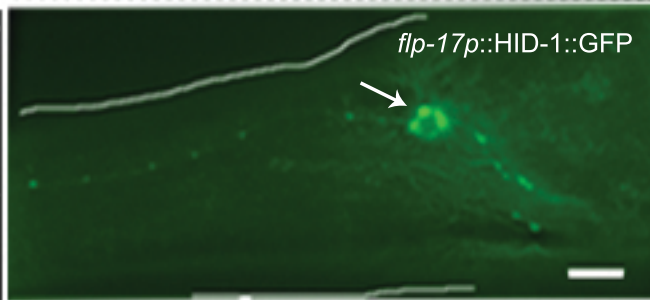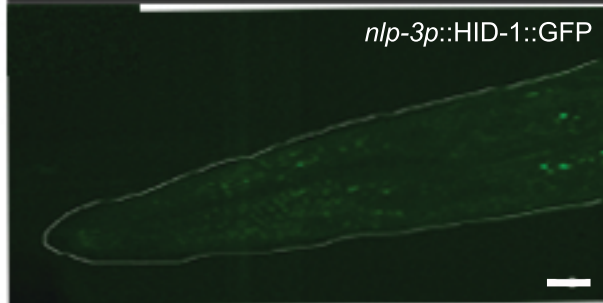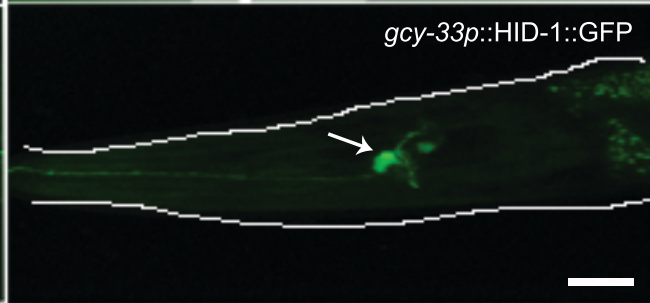

Supplement: Figure S3 — Transgenic expression of HID-1::GFP. HID-1 fused to eGFP was expressed under its own promoter in the background of yg316 or under gcy-8, nlp-3, osm-6, flp-17, or gcy-33 promoters in the background of sa722. Arrows indicate the AFD neurons (gcy-8p) and BAG neurons (flp-17p and gcy-33p).The expression of hid-1p, nlp-3p and osm-6p was detected in several neurons. Scale bar, 10 µm. (PDF) [file pgen.1004529.s003.pdf]

Figure S4

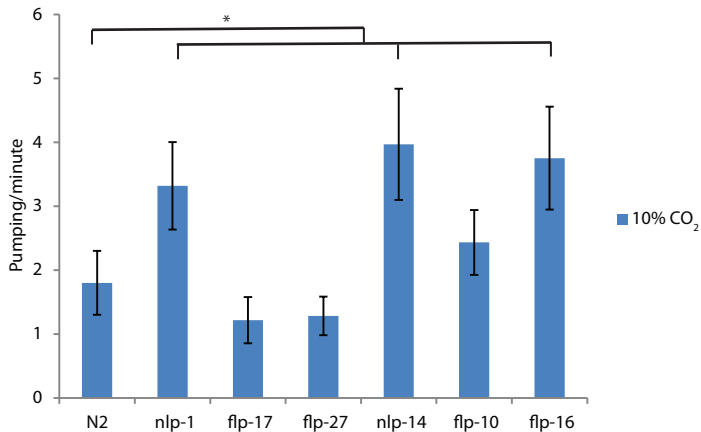

Supplement: Figure S4 — Animals with deletions in neuropeptide genes expressed in the BAG neurons still show strong CO2-mediated pumping inhibition. One-day-old animals with mutations in neuropeptide genes, which are known to be overexpressed in the BAG neurons, were exposed to 10% CO2 and pumping rate was measured. The pumping rate of nlp-1, nlp-14, and flp-16 mutants in 10% CO2 (but not in normal air conditions) was significantly different from that of the wild-type (N2) animals and showed small but significant rescue. *P<.01. Error bars indicate SEM. (PDF) [file pgen.1004529.s004.pdf]
